# Long-term effects of maize straw return and manure on the microbial community in cinnamon soil in Northern China using 16S rRNA sequencing

**DOI:** 10.1371/journal.pone.0249884

**Published:** 2021-04-22

**Authors:** Zhiping Liu, Huaiping Zhou, Wenyan Xie, Zhenxing Yang, Qianqian Lv

**Affiliations:** 1 College of Resources and Environment, Shanxi Agricultural University, Taiyuan, P. R. China; 2 College of Biological Engineering, Shanxi University, Taiyuan, P. R. China; University of Salento, ITALY

## Abstract

Excessive use of chemical fertilizers in agricultural practices have demonstrated a significant impact on microbial diversity and community in soil by altering soil physical and chemical properties, thereby leading to a certain degree of soil salinization and nutritional imbalances. As an organic amendment, maize straw has been widely used to improve soil quality; however, its effect on the soil bacterial community remains limited in Calcarie-Fluvie Cambisols soil in semi-humid arid plateau of North China. In the present experiment, we investigated the effects of continuous straw utilization and fertilization on bacterial communities in Shouyang, Shanxi province, China. Soil samples were collected from 5 different straw utilization and fertilization modes in the following ways: straw mulching (SM), straw crushing (SC), cattle manure (CM), in which way straw is firstly used as silage and then organic fertilizer, control with no straw return (NSR), and control without fertilizers (CK), same amount of N+P fertilizer was applied to the regimes except CK. High-throughput sequencing approaches were applied to the V3-V4 regions of the 16S ribosomal RNA for analysis of the bacterial abundance and community structures. Different long-term straw returning regimes significantly altered the physicochemical properties and bacterial communities of soil, among which CM had the most significant effects on soil fertility and bacterial diversity. *Proteobacteria*, *Actinobacteria*, *Chloroflexi*, *Acidobacteria*, and *Gemmatimonadetes* were consistently dominant in all soil samples, and Redundancy analysis (RDA) showed significant association of total nitrogen (TN), total phosphorus (TP) and available potassium (AK) with alternation of the bacterial community. Cattle manure had the most beneficial effects on soil fertility and bacterial diversity among different straw utilization and fertilization modes.

## Introduction

As an integral part of the soil, microorganisms represent the major driving force of soil organic matter and nutrient cycling, playing a key role in soil nutrient conversion, energy transformation, and the formation of soil organic matter [1–4]. Due to the rapid response of bacteria to changes in the soil environment, bacterial communities were selected as early bio-indicators of soil quality [5]. Bacterial diversity and community composition are affected by soil pH, organic matter, soil moisture content, mineral substances etc. [6, 7], which could be influenced by fertilization management practices [8, 9].

As the largest grain producer worldwide, agriculture production has largely depended on high inputs of chemical fertilizer, while about 800 million tons of crop straw are annually produced in China [10]. Crop straw has complex components, such as high contents of lignin, cellulose, and hemicellulose, which is rich in organic carbon, nitrogen, phosphorus, potassium, silicon, and other mineral nutrients [11–13], and has been widely utilized in the field to promote soil carbon sequestration and consequently maintaining soil fertility. Previous studies have documented the positive and significant impact of straw utilization on soil microbial community structure, biomass, and activity [14–18], leading to increases in soil granular structure and the content of water-stable aggregates for better soil fertility and water conservation [19–21]. Chen et al. documented a significant increase in total phospholipid fatty acids (PLFA), bacterial biomass, and actinomycete biomass under short-term straw return in Jiangyan, China [5]. Zhao et al. reported an abundant increase of Gram-negative (Gm-) bacteria, but only after long-term maize straw returned (30 years) in a summer maize-winter wheat cropping system, one located in north-central China [13].

The Shanxi province is a semi-humid arid plateau of North China with a typical soil of Calcarie-Fluvie Cambisols. Under the local soil type and climate conditions, we hypothesized that different maize straw utilization and fertilization would affect the diversity and community structure of soil bacteria. However, the understanding of the effects of straw utilization and fertilization on bacterial communities in this region remains limited. Considering the imperfection of traditional cultivation on growing media, high-throughput sequencing approaches were applied to the V3-V4 regions of the 16S ribosomal RNA for analysis of soil bacteria to different maize straw utilization and fertilization modes. Five treatments were applied: (1) straw mulching (SM); (2) straw crushing (SC); (3) cattle manure (CM), in which way maize straw is firstly used as silage, and cattle manure is applied to field as organic fertilizer; (4) control with no straw return (NSR); and control without fertilizers (CK) same amount of N+P fertilizer was applied to the regimes except CK. Soil samples were collected for analysis of bacterial diversity. The objectives of this study were to: (1) elucidate the influences of different straw utilization and fertilization modes on bacterial diversity and community structure; (2) search for the most suitable straw utilization mode.

## Materials and methods

### Experimental design

The long-term straw utilization and fertilization experiment was established in Shouyang, Shanxi province, China (28°15′20″N, 116°55′30″E) in 1992. The area belongs to the continental monsoon climate in a mid-latitude, temperate-warm, and semi-humid, arid area, featuring four distinct seasons. The average annual temperature is 7.4°C with a frost-free period of about 130 days, whereas the average annual precipitation is 500 mm with an annual evaporation of 1600–1800 mm. The experimental site has a sandy loam cinnamon soil, classified as a Calcarie-Fluvie Cambisols in WRB [22, 23]. The soil layer is deep, and the groundwater level is 50 meters below the surface. Analysis of soil samples taken from the experimental area showed that the basic physical and chemical properties of the surface 15 cm of soil were as follows: pH, 8.4; organic matter (OM), 23.8 g/kg; available N (AN), 117.69 mg/kg; available P (AP), 4.84 mg/kg; and available K (AK), 100 mg/kg. The authority who issued the permission for each location is “Shanxi Shouyang Field Scientific Observation and Experimental Station”.

The long-term trial began in the spring of 1992, lasting 27 years. The five treatments were arranged in a randomized block design with three replications, with each plot being 67 m^2^. The five treatments were (1) SM, straw mulched on the soil in the first year and then crushed in the second year to incorporate into the soil; (2) SC, straw crushed directly to incorporate into the soil; (3) CM, cattle manure, maize straw is firstly used as silage, and cattle manure is applied to field as organic fertilizer; (4) NSR, control, no straw using, same amount of N+P fertilizer was applied to the above regimes and (5) CK, control without fertilizers. Straw in treatments SM, SC, and CM had an equivalent amount of carbon. The fertilization details are shown in Table 1. All the chemical fertilizers and straws were applied as basal fertilizers before planting.

**Table 1:** Experimental treatments and fertilization.

Treatments	Inorganic fertilizer (kg/hm^2^)			Straw (kg/hm^2^)			Cattle Manure (kg/hm^2^)			Total nutrient (kg/hm^2^)		
	N	P_2_O_5_	K_2_O	N	P_2_O_5_	K_2_O	N	P_2_O_5_	K_2_O	N	P_2_O_5_	K_2_O
SM	150	84	0	46	11	82	0	0	0	196	95	82
SC	150	84	0	46	11	82	0	0	0	196	95	82
CM	150	84	0	0	0	0	350	236	756	500	320	756
NSR	150	84	0	0	0	0	0	0	0	150	84	0
CK	0	0	0	0	0	0	0	0	0	0	0	0

SM: straw mulched; SC, straw crushed; CM, cattle manure; NSR, fertilizer with no straw return; and CK, no fertilizer and no straw return.

### Soil sampling and analysis of soil properties

Soil was randomly sampled from 5 points in each plot on October 1, 2018 and mixed to form one composite sample per plot. Each mixed soil sample was placed in sterile bag and transported to a laboratory in an icebox. After removing visible stones and plant debris, one part was stored at -80°C for DNA extraction the other part was gently broken along its natural breakpoints, sieved through a 2 mm screen, thoroughly mixed for physicochemical properties analysis.

### Soil physicochemical analytical procedures

Soil total nitrogen (TN), total phosphorus (TP), and total kalium (TK) were analyzed using a Vario Max element analyzer (Elementar Vario PYRO cube and Isoprime100, Germany). Soil pH was determined with a soil (air-dried) to water (w/w) ratio of 1:2.5 with a pH meter. OM was determined by K_2_Cr_2_O_7_ oxidation method. Soil AN was determined by the alkaline hydrolysis diffusion method. AP was determined by NaHCO_3_-extracted method. AK was analyzed by CH_3_COONH_4_-extracted method.

### Soil DNA extraction and high-throughput sequencing

Total DNA was extracted from 0.5 g soil of each sample using the Fast DNA SPIN Isolation Kit (MP Biomedicals, Santa Ana, CA, United States) according to the manufacturer’s instructions. The quantity and quality of DNA were accessed using a NanoDrop ND-1000 Spectrophotometer (Thermo Fisher Scientific, Waltham, MA, USA) and on 0.8% agarose gel electrophoresis, respectively.

The bacterial V3-V4 hypervariable region of 16S rRNA was amplified using primers 338F (5’-ACTCCTACGGGAGGCAGCA-3’) and 806R (5’-GGACTACHVGGGTWTCTAAT-3’). Specific barcodes of 7-bp were incorporated into the primers for multiplex sequencing. PCR reactions were performed in a system of 25 μL mixture containing 5 μL of 5×Q5 Reaction Buffer, 5 μL of 5×Q5 High-Fidelity GC Buffer, 2 μL of dNTPs (5U/μL), 1 μL of each primer, 0.25 μL of Q5 High-Fidelity DNA Polymerase (Transgen, China), 2μL of temple DNA, and 8.75 μL of ddH_2_O.

PCR was conducted as follows: 2 min of initial denaturation at 98°C, followed by 25 cycles of denaturation at 98°C for 15 s, annealing at 55°C for 30 s and extension at 72°C for 30 s, and a final extension of 5 min at 72°C. PCR amplicons were purified with Agencourt AMPure Beads (Beckman Coulter, Indianapolis, IN) and quantified using the PicoGreen dsDNA Assay Kit (Invitrogen, Carlsbad, CA, USA). After purification and being pooled at equal concentrations, the PCR products were pair-end sequenced with 2*300 bp on the Illumina MiSeq platform with MiSeq Reagent Kit v3 at Personal Biotechnology (Shanghai, China).

### Bioinformatics analysis

Raw sequencing data was processed with Quantitative Insights Into Microbial Ecology (QIIME, v1.8.0) software [24]. Raw sequencing reads with exact matches to the barcodes were assigned to respective samples and identified as valid sequences. The low-quality sequences were filtered and paired-end reads were assembled using FLASH [25]. After chimera detection, the high-quality sequences were clustered with UCLUST [26] into operational taxonomic units (OTUs) at 97% similarity. A representative sequence was selected from each OTU using default parameters. OTU taxonomic classification was conducted by BLAST searching the representative sequences set against the Silva 132 Database [27] using the best hit [28]. An OTU table was generated to record the abundance and taxonomy of each OTU in each sample. OTUs with less than 0.001% of total sequences across all samples were discarded. To minimize the difference of sequencing depth across samples, an averaged, rounded, and rarefied OTU table was generated by averaging 100 evenly resampled OTU subsets under the 90% of the minimum sequencing depth for further analysis.

Sequence data analyses were mainly performed using QIIME and R packages. OTU-level α-diversity indices, such as Chao1 richness estimator, ACE metric (Abundance-based Coverage Estimator), Shannon diversity index, and Simpson index were calculated using the OTU table in QIIME. OTU-level ranked abundance curves were generated to compare the richness and evenness of OTUs among samples. β-diversity analysis was performed to investigate the structural variation of microbial communities across samples, and nonmetric multidimensional scaling (NMDS) based on OTU was used [29]. The significance of differentiation of microbiota structure among groups was assessed by permutational multivariate analysis of variance (PERMANOVA) [30] and analysis of similarities (ANOSIM) [31, 32]. The taxonomy compositions and abundances were visualized using MEGAN [33]. A Venn diagram was generated to visualize the shared and unique OTUs among samples or groups based on the occurrence of OTUs across groups, and regardless of their relative abundance [34]. Taxa abundances at the phylum, class, order, family and genus levels were statistically compared among samples by Metastats [35]. Linear discriminant analysis (LDA) effect size (LEfSe) was performed to detect differentially abundant taxa across groups using the default parameters [36].

### Statistical analysis

A one-way analysis of variance (ANOVA) was performed to determine the effects of different straw utilization and fertilization regimes on soil physicochemical characteristics with Microsoft Excel (Microsoft Corporation, USA) and SPSS Windows version 11.0 (SPSS Inc., Chicago, USA) packages. Significant differences between soil physicochemical characteristics were determined with the least significant difference (LSD) test at the p = 0.05 level.

## Results

### Soil physicochemical characteristics

Compared with no straw return (NSR) and no fertilizer (CK), all nutrient indices including the contents of total nitrogen (TN), total phosphorus (TP), total kalium (TK), available N (AN), available P (AP), available K (AK), and organic matter (OM) were significantly increased (*p*<0.05) except soil pH, indicating that significant changes in soil chemical properties were caused by the long-term straw return. All nutrient indices were the highest in CM treatment, followed by SC treatment, SM treatment, and NSR treatment. Soil pH (H_2_O) values were significantly decreased by the addition of crushed straw (SC) and cattle manure (CM), as well as by no straw returning (NSR), compared with CK and mulching straw (SM). The pH in CM treatment was more neutral compared with other treatments. The contents of AN and TN in SM treatment were significantly lower than in SC treatment; however, AP, AK, OM, and TK were significantly higher in SM treatment than in SC treatment (Table 2).

**Table 2:** Soil pH and nutrient contents in a utilization and fertilization experiment.

Treatments	pH	AN (mg/kg)	AP (mg/kg)	AK (mg/kg)	OM (g/kg)	TN (g/kg)	TP (g/kg)	TK (g/kg)
SM	8.66±0.12a	40.95±0.05c	32.97±0.02b	85.06±0.02b	30.43±0.01b	1.10±0.01c	0.74±0.03b	16.19±0.08b
SC	8.52±0.02b	45.05±0.06b	32.25±0.04c	78.69±0.12c	28.28±0.02c	1.20±0.02b	0.74±0.12b	15.37±0.12c
CM	8.32±0.05c	72.31±0.13a	73.72±0.12a	124.34±0.05a	36.02±0.12a	1.42±0.31a	0.93±0.22a	20.89±0.13a
NSR	8.54±0.01b	38.9±0.09d	28.88±0.05d	50.02±0.06d	24.62±0.31d	1.02±0.09d	0.63±0.07c	15.04±0.01d
CK	8.65±0.10a	32.76±0.12e	4.56±0.03e	21.68±0.04e	12.90±0.07e	0.80±0.10e	0.50±0.29d	14.67±0.09e

AN: available N; AP: available P; AK: available K; OM: organic matter; TN: total N; TP: total P; TK: total K; Different letters in the same column indicate a significant difference (ANOVA followed by Turkey-Kramer post hoc test, n = 3, p<0.05, average value, SD standard deviation).

### Bacterial alpha-diversity

A total of 376,833 clean sequences were generated and deposited in the NCBI Sequence Read Archive (PRJNA588667). The number of species observed in the sample was not significantly increased with the addition of new samples (S1 Fig), indicating that the sample size employed was large enough to characterize the bacterial communities.

The diversity indices Chao1 estimator, abundance-based and coverage estimator (ACE), Shannon index (Hʼ), and the Simpson index (D) were estimated as community richness indices using a randomly selected subset of about 12000 sequences per soil sample. As shown in Table 3, the diversity indices were the lowest in CK but the highest in CM treatment. However, no significant differences in bacterial richness or diversity were detected among different treatments, as indicated by Chao1, H’, ACE, and D indices.

**Table 3:** Richness and alpha-diversity indices of the different OTUs under the different treatments.

Treatments	Chao1		ACE		Shannon		Simpson	
	Mean	SD	Mean	SD	Mean	SD	Mean	SD
SM	3144.86a	274.72	3320.63a	327.77	10.76a	0.05	0.99883a	6.7E-05
SC	3051.50a	309.06	3233.18a	388.58	10.76a	0.06	0.99891a	7.27E-05
CM	3148.42a	442.18	3334.90a	473.97	10.76a	0.02	0.99897a	2.35E-05
NSR	3122.67a	354.57	3273.54a	434.61	10.80a	0.02	0.99888a	1.47E-05
CK	2931.36b	482.38	3019.21a	594.90	10.71a	0.08	0.99863a	9.76E-05

Different letters in the same column indicate a significant difference (ANOVA followed by Turkey-Kramer post hoc test, n = 3, p<0.05, average value, SD standard deviation).

### Soil bacterial community composition

**Fig 1:**
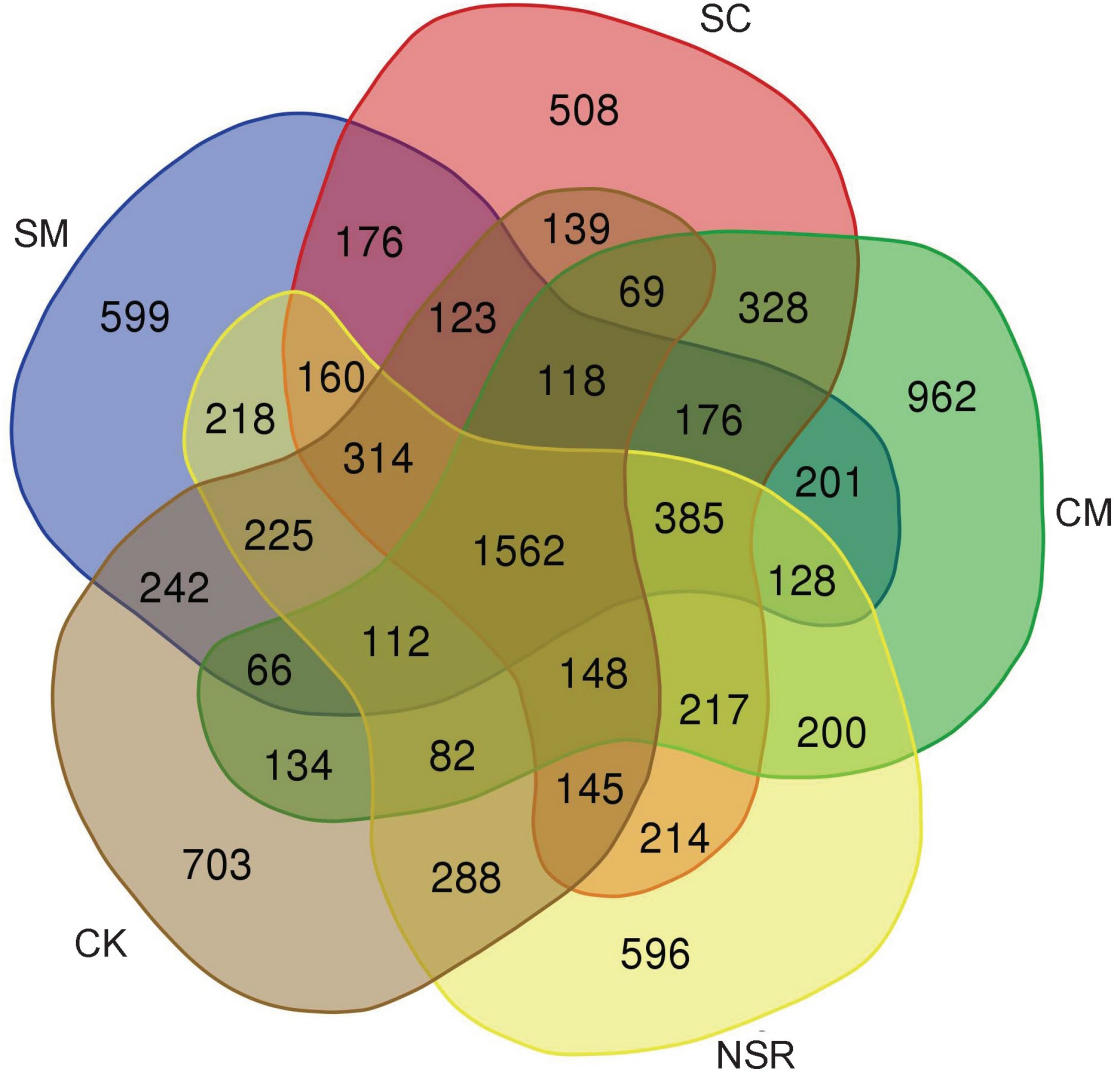
Venn diagram showing unique and overlapped OTUs between different straw return treatments.

As shown in Fig 1, 1562 OTUs were shared by different treatments. The number of OTUs specific to treatment SM, SC, CM, NSR, and CK were 599, 508, 962, 596, and 703, respectively. Compared with treatment SM, SC, NSR, and CK, unique OTUs in treatment CM were increased by 60%, 89%, 61%, and 37%, respectively.

### Effects of different treatments on soil bacterial communities

**Fig 2:**
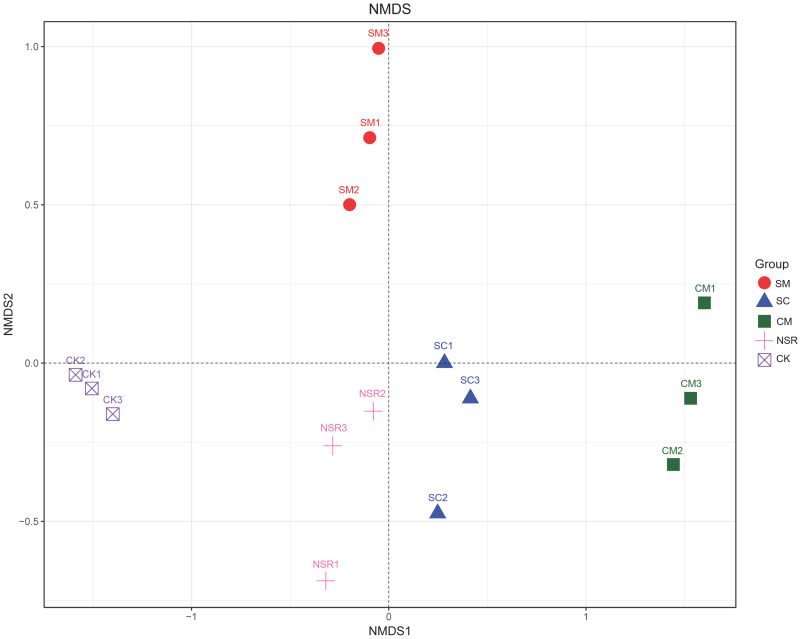
Nonmetric multidimensional scaling of the bacterial community compositions in soil under different treatments based on OTUs.

Nonmetric multidimensional scaling (NMDS) was used to compare the similarity of soil bacterial communities among different treatments at the OTU level (Fig 2). The three repeats of each of the treatments were clustered together, showing good repeatability. The samples of treatment CM were located in the right part of the graph, whereas the samples of CK were gathered at the left and samples of treatment SM were gathered in the upper part. Samples of SC and NSR distributed separately along the vertical axis, which were relatively closer among all the treatments. The results showed a significant difference in the bacterial community structures among treatment SM, CM, and CK at the OTU level.

### Taxonomic composition analysis at the phylum and genus level

**Fig 3:**
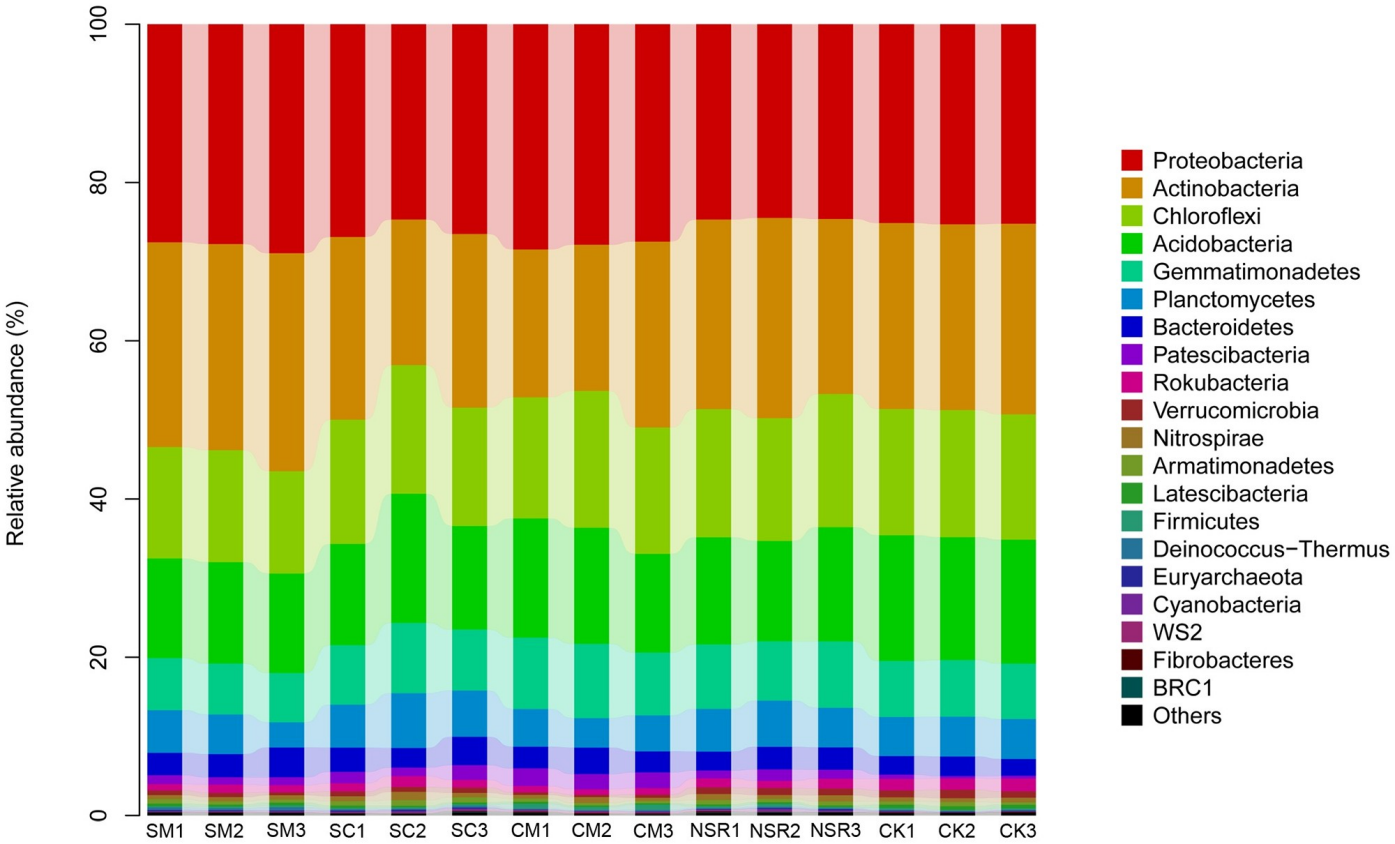
Taxonomic composition and abundance distribution of bacteria at the phylum level under different treatments.

**Fig 4:**
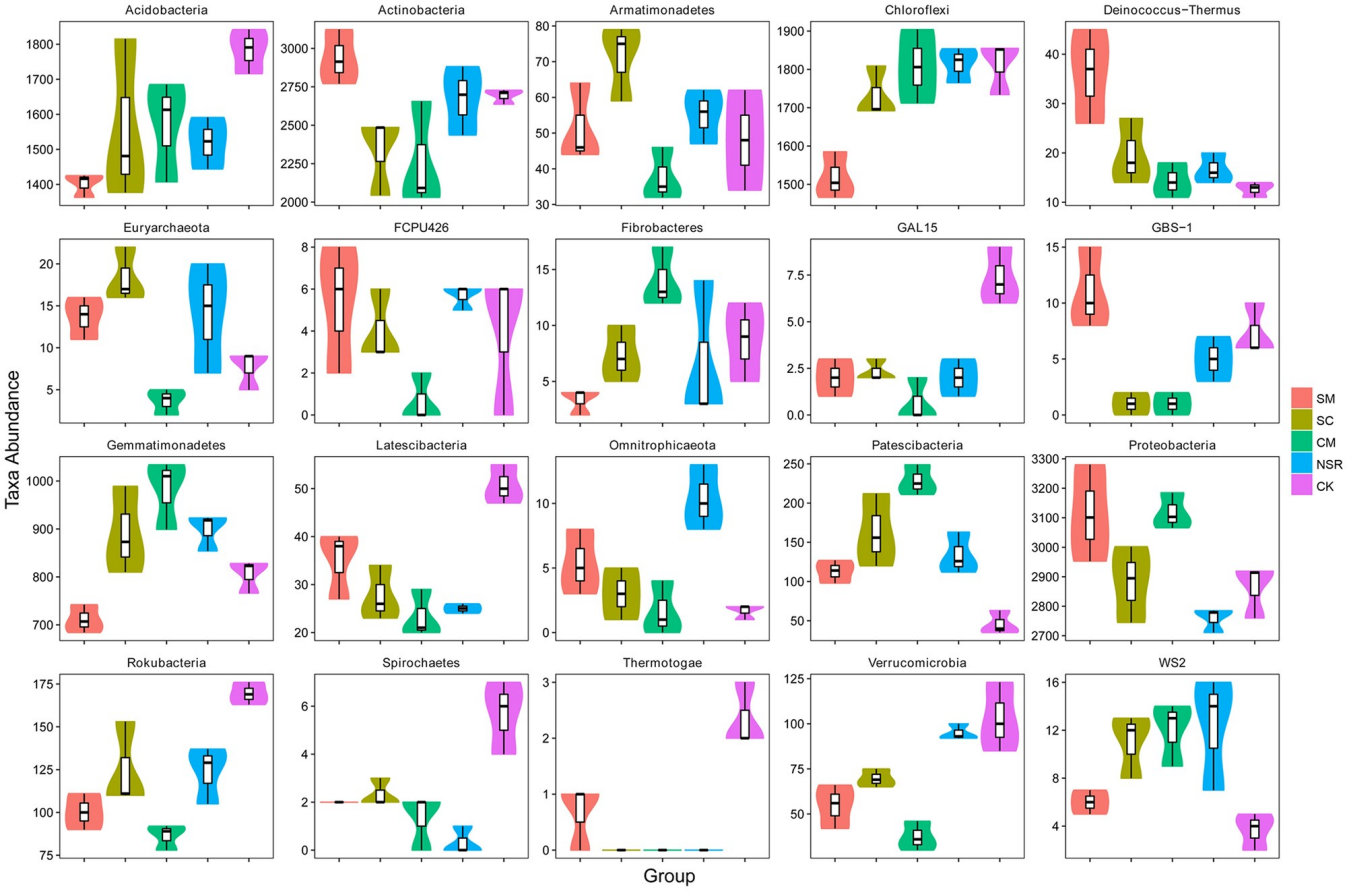
The top 20 phyla with significant differences between the five treatments.

The taxonomic distributions of bacterial communities were evaluated at different levels of classification. As shown in Fig 3, *Proteobacteria*, *Actinobacteria*, *Chloroflexi*, *Acidobacteria*, *Gemmatimonadetes*, *Planctomycetes*, *Bacteroidetes*, *Patescibateria*, *Rokubacteria*, and *Verrucomicrobia* were the most abundant at the phylum level, accounting for approximately 97.5% of the microorganisms detected in all 15 soil samples. Compared with treatment NSR, the percentage of *Proteobacteria* was increased by 3.50% in treatment SM, 1.43% in treatment SC, and 3.33% in treatment CM. The top 20 phyla with significant differences among all the treatments are shown in Fig 4. Among the top 5 phyla, *Proteobacteria*, *Chloroflexi*, *Acidobacteria*, and *Gemmatimonadetes* increased by 3.33%, 0.02%, 0.51%, and 0.78% in treatment CM, respectively.

At the genus level, the dominant genera were *Sphingomonas*, *MND1*, *RB41*, *AKYG587*, *Nocardioides*, *Blastococcus*, *Micromonospora*, *Actinoplanes*, *Haliangium*, and *Allorhizobium-Neorhizobium-Pararhizobium-Rhizobium* (S2 Fig). The relative abundance of *Sphingomonas* was 1.13% in treatment SM, 0.42% in treatment SC, and 0.09% in treatment CM, as compared to treatment NSR. The percentage of *MND1* increased by 0.46% in treatment SM, 0.22% in treatment SC, and 0.13% in treatment CM, as compared with treatment NSR. The top 20 genera with a significant difference among all the treatments are shown in S3 Fig. The taxonomic composition and abundance distribution of bacterias at the class, order, family level under different treatments are shown in S4–S6 Figs in the supporting information.

LEfSe revealed 7, 1, 6, 0, and 1 taxa with significant differences (LDA score>4, and *p<0*.*05*) among five treatments (S7 Fig), which might serve as biomarkers for different treatments.

### Heat map of community composition combined with cluster analysis

**Fig 5:**
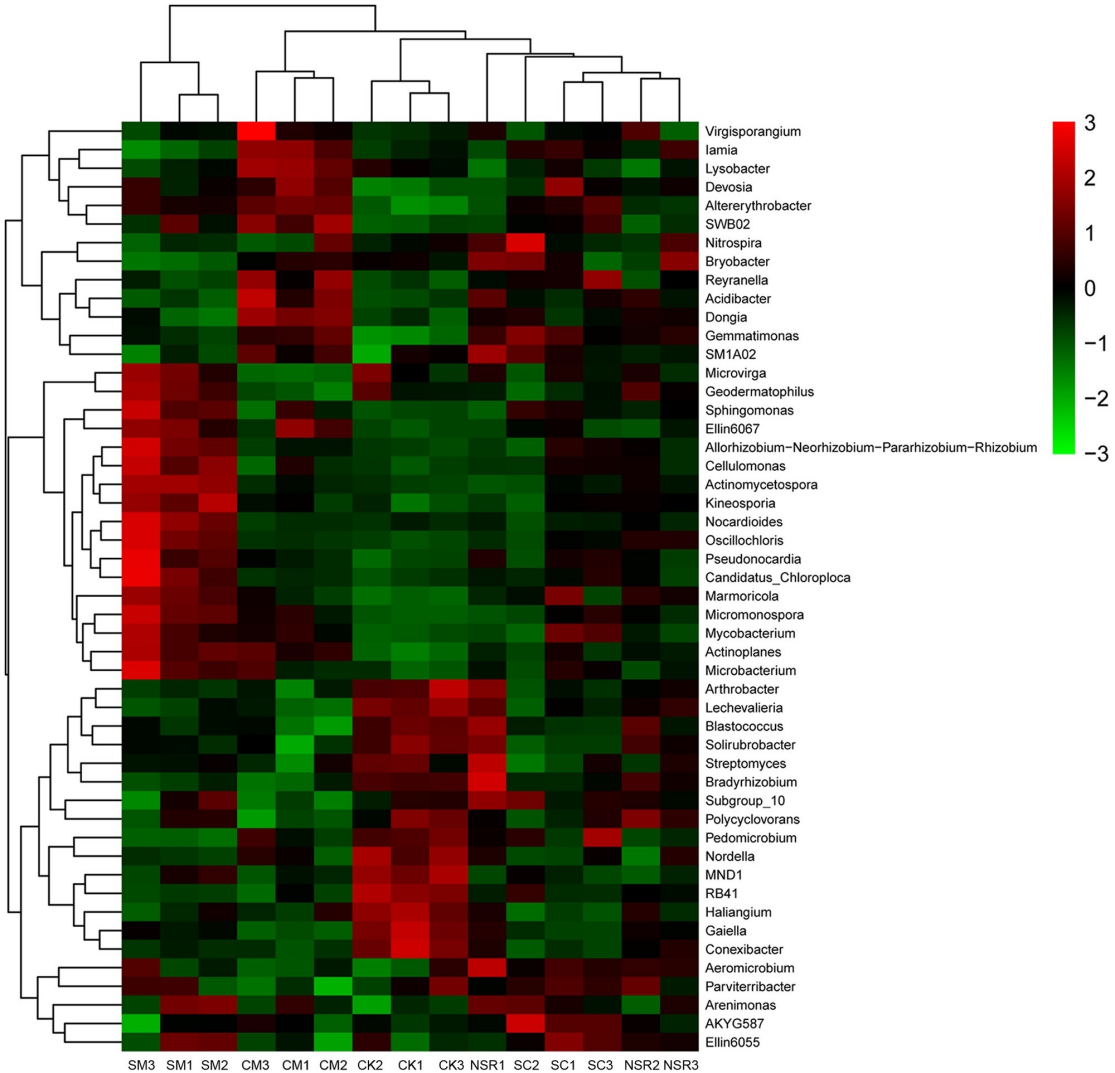
Hierarchically clustered heat map analysis of community composition in the soil samples under different treatments.

The genera with the top 50 abundance were clustered, showing large variation among the five treatments (Fig 5). Genera *Nocardioides*, *Oscillochloris*, *Pseudonocardia*, *Candidatus-Chloroploca*, and *Microbacterium* were relatively higher in abundance in treatment SM, whereas *Virgisporangium*, *Lamia*, *Lysobacter*, *Devosia*, and *SWB02* were relatively higher in abundance in treatment CM.

### Relationship between soil bacterial communities and environmental variables

**Fig 6:**
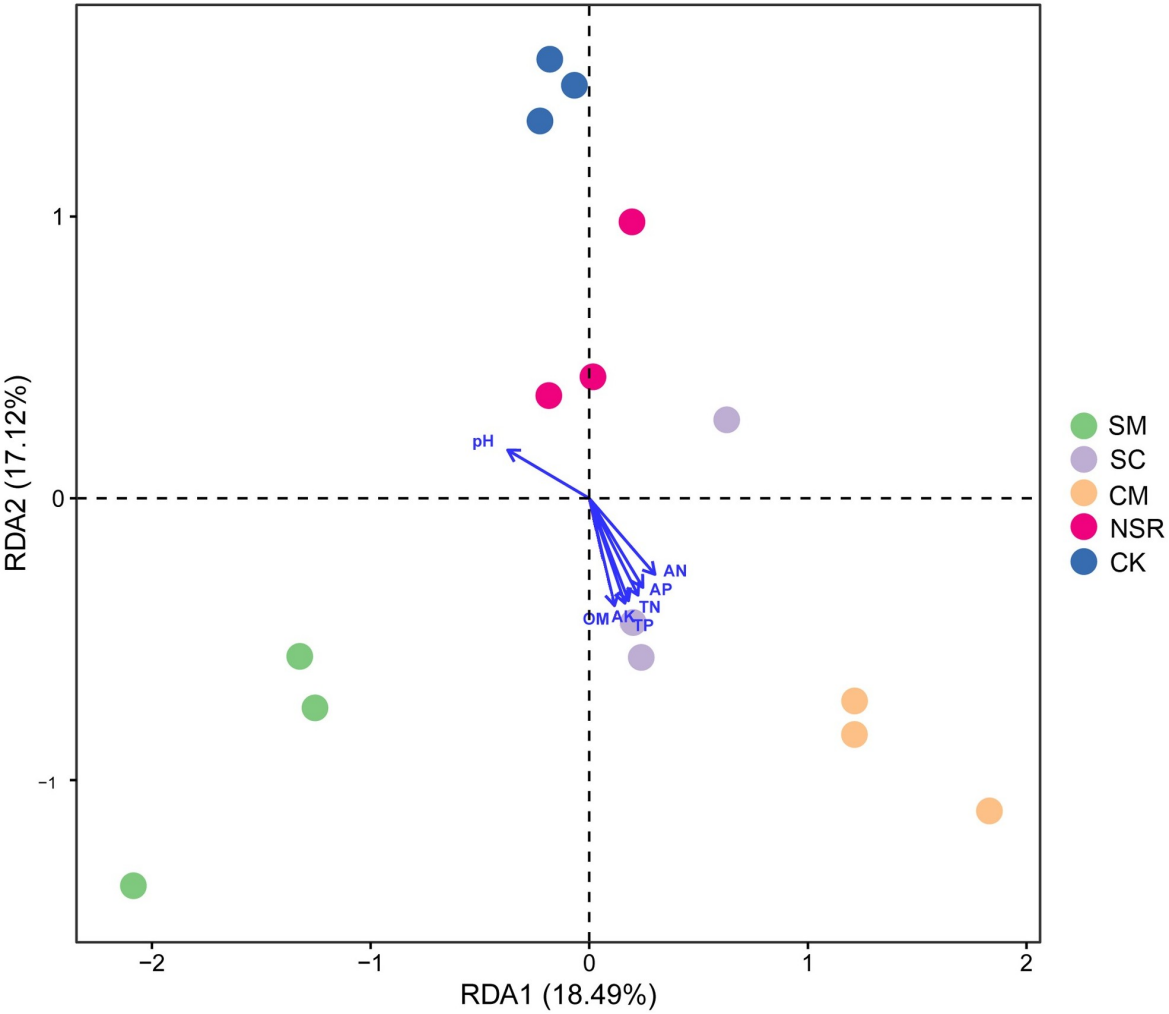
Redundancy analysis between soil samples based on OTUs.

Redundancy analysis (RDA) was conducted to explore the possible physicochemical properties (pH, OM, TP, TN, TK, AP, AN, AK) differentiating soil bacterial community structures. As shown in Fig 6, eight environmental variables (pH, OM, TP, TN, TK, AP, AN, AK) together explained 35.61% of the total variance. The first two RDA axes explained most of the variation, in which the first axis accounted for 18.49% and the second axis explained 17.12% of the variation (Fig 6). The Monte Carlo permutation test (S1 Table) revealed that TN, TP and AK were the main environmental factors affecting soil bacterial distribution.

## Discussion

### Effects of straw return on the soil chemical properties

The soil bacterial communities generally change when nutrients are added to the soil. In the present study, treatment CK had not added fertilizers for 27 years, leading to the lowest chemical properties. Compared with CK and NSR, different straw utilization and fertilization regimes significantly improved the soil chemical properties including OM, TP, TN, TK, AP, AN, and AK, among which treatment CM performed the best. However, treatment CM reduced soil pH, consistent with the findings of Liu et al., who reported a decline in soil pH with the application of manure, resulting in an increased risk of soil acidification [37]. The reason might be that cattle manure contains a large amount of nutrients, particularly AN. Moreover, the contents of AP, AK, OM, and TK were significantly higher in treatment SM than in treatment SC, which might be caused by straw that was decomposed in treatment SM, as opposed to the fresh straw in treatment SC. Notably, the soil pH had no significant difference between the straw mulching and CK over 27 years. Previous studies demonstrated that decomposition facilitated the release of nutrients of the straw [38, 39]. SM has been proven to improve soil water retention, thereby enhancing soil moisture conservation under arid and semiarid conditions [17, 40] to promote plant growth [41].

### Effects of straw utilization and fertilization on the soil bacterial community

Consistent with the previous results reported in the wheat-rice rotation system [42], alpha diversity analysis showed no significant effects of long-term straw utilization and fertilization on bacterial community richness and diversity. NMDS showed significant differences (*p<0*.*05*) in bacterial community structure under different treatments, which is consistent with the results of Bei et al., who documented clear separation of bacterial and fungal community composition between the straw utilization and no straw utilization [43]. Therefore, straw utilization directly affects the structure of soil microorganisms by promoting or inhibiting the change of soil microorganism composition, further impacting the biological activity of microorganisms in the soil. Among different straw utilization and fertilization regimes, treatment CM showed the most significant effect on bacterial community structure.

Maize straw is composed by cellulose (35%), hemicellulose (27%), lignin (12%), and other complex substances, and is mainly decomposed by soil microorganisms in two phases. Our study demonstrated no significant effect of different long-term maize straw utilization and fertilization regimes on the bacterial community structure at the phylum level, which is consistent with the results of Xia et al., who reported no significant change of bacterial community structure with straw using [44]. *Proteobacteria*, *Actinobacteria*, *Chloroflexi*, *Acidobacteria*, and *Gemmatimonadetes* were the dominant phyla in our study, accounting for approximately 86.7%, which is consistent with the previous results reported in maize planting land, wheat-maize rotation system [43], forest, vineyard, copper mine, river, lake, and greenhouse [45–50]. Among these phyla, *Proteobacteria* and *Gemmatimonadetes* mainly participated in the initial stage of straw decomposition, whereas *Actinobacteria* is involved in almost all of the straw degradation phase. The addition of straw changes the soil pH, thereby favoring the growth of *Actinobacteria*. *Chloroflexi* is generally believed to produce energy through photosynthesis and have a certain degradation function for soil environmental pollution [51].

Under long-term straw utilization and fertilization conditions, *Sphingomonas*, *MND1*, *RB41*, *AKYG587*, *Nocardioides*, *Blastococcus*, *Micromonospora*, *Actinoplanes*, *Haliangium*, and *Allorhizobium-Neorhizobium-Pararhizobium-Rhizobium* together occupied the dominant position. *Sphingomonas* was one of the most effective microorganisms for degradation of soil toxic substances, promotion of nutrient absorption and resistance to multiple pathogens. Previous studies reported several *Sphingomonas* strains with the characteristics of nitrogen fixation and dehydrogenation, playing an important role in maintaining the nitrogen balance of soil [52]. In addition to the dominant known bacteria, unclassified bacteria still accounted for a certain proportion in maize soil. Further study is needed to characterize unclassified bacteria and their function in long-term straw-using maize soil.

The LEfSe analysis demonstrated that *Gemmatimonadetes* (*Gemmatimonadetes*, *Gemmatimonadales* and *Gemmatimonadetes*) and *Chloroflexi (Anaerolineae* and *SBR1031*) might serve as indicators for treatment CM. *Gemmatimonadetes* were ubiquitous in soil, comprising ~2% of soil bacterial communities [53] and playing an important role in terrestrial ecosystems. However, the distribution of *Gemmatimonadetes* was more dependent on moisture availability in soil, and it was unable to resist moisture fluctuations induced by wet/dry cycling [53]. Additionally, the distribution of *Gemmatimonadetes* was also constrained by soil. *Chloroflexi* was distributed widely in terrestrial and aquatic ecosystems [54], whereas *Anaerolineae* was abundant in active sludge and other nutrient rich environments [55]. Among different straw utilization and fertilization regimes, long-term cattle manure was shown to be the most favorable for the growth and distribution of these bacteria.

## Conclusion

In the present study, soil under continuously long-term straw utilization and fertilization were used to characterize physicochemical properties and bacterial communities. The results indicated that different straw utilization and fertilization had significant effects on soil properties and bacterial communities. Among different straw utilization regimes, cattle manure, which was used as silage first and then organic fertilizer, showed the richest nutrients and highest bacterial diversity. Therefore, cattle manure appears to be the most promising agricultural practice for the improvement of soil fertility.

## Supporting information

S1 Fig Species accumulation curve of bacteria. (TIF)

S2 Fig Taxonomic composition and abundance distribution of bacteria at the genus level under different treatments. (TIF)

S3 Fig The top 20 genera with significant differences between the five treatments. (TIF)

S4 Fig Taxonomic composition and abundance distribution of bacteria at the class level under different treatments. (TIF)

S5 Fig Taxonomic composition and abundance distribution of bacteria at the order level under different treatments. (TIF)

S6 Fig Taxonomic composition and abundance distribution of bacteria at the family level under different treatments. (TIF)

S7 Fig Taxons with significant difference between treatments. (TIF)

S1 Table The Monte Carlo permutation test. (DOCX)
