# Supplementary figures and images for: Long-term effects of maize straw return and manure on the microbial community in cinnamon soil in Northern China using 16S rRNA sequencing
Source: PLoS One. 2021 Apr 22;16(4):e0249884. doi: 10.1371/journal.pone.0249884 (PMC8062091; doi:10.1371/journal.pone.0249884)

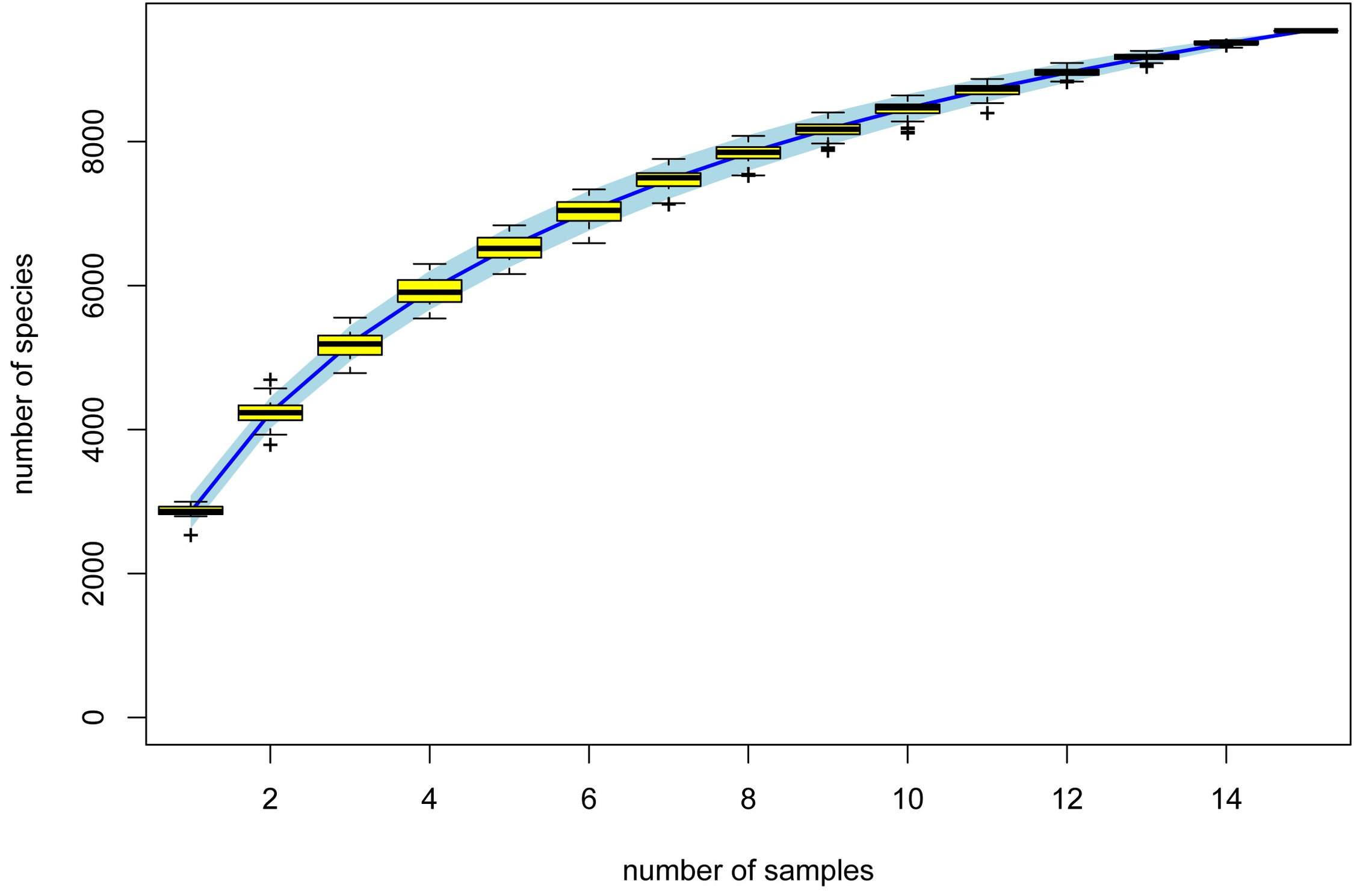

Supplement: S1 Fig — (TIF) [file pone.0249884.s001.tif]

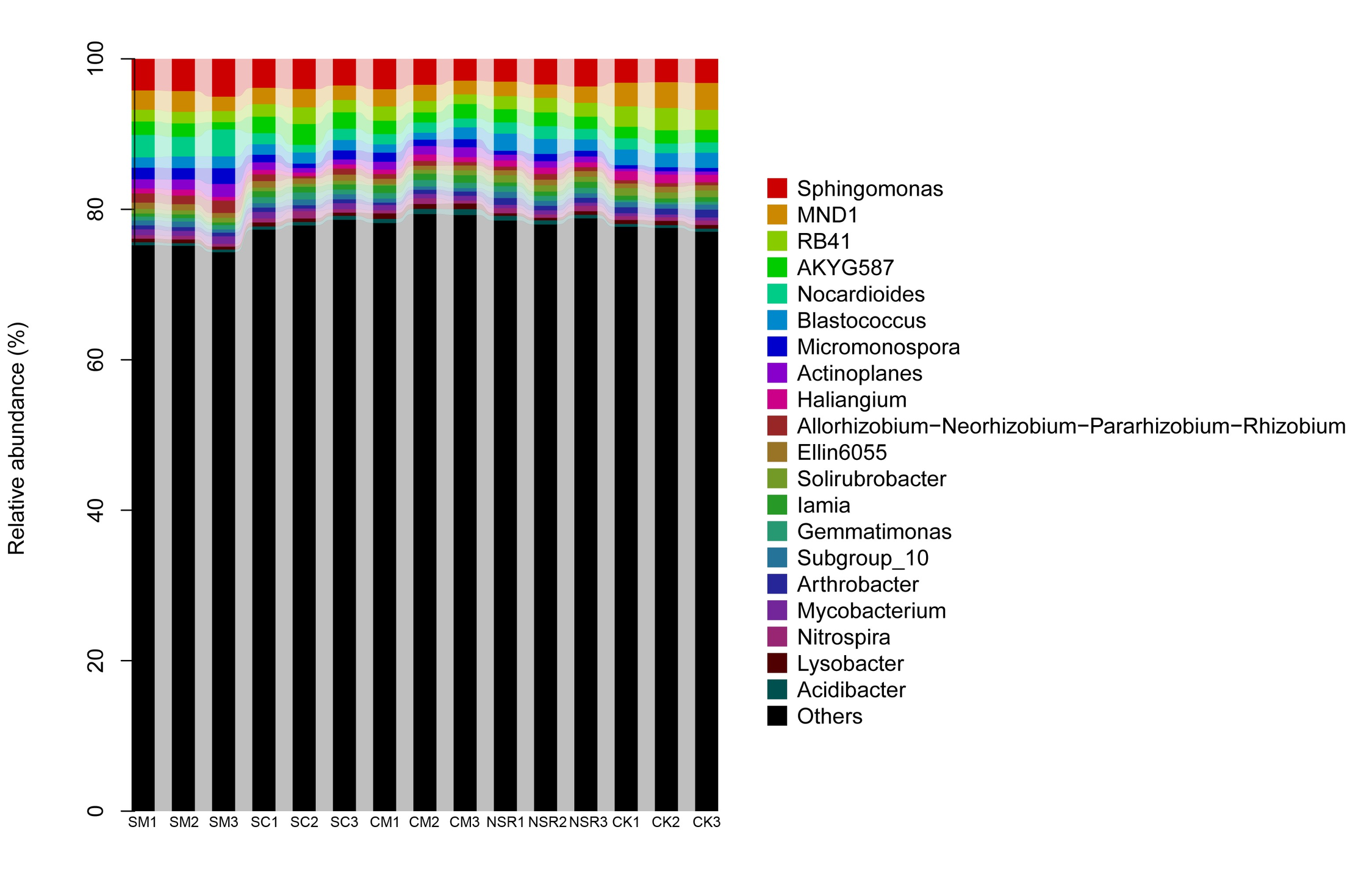

Supplement: S2 Fig — (TIF) [file pone.0249884.s002.tif]

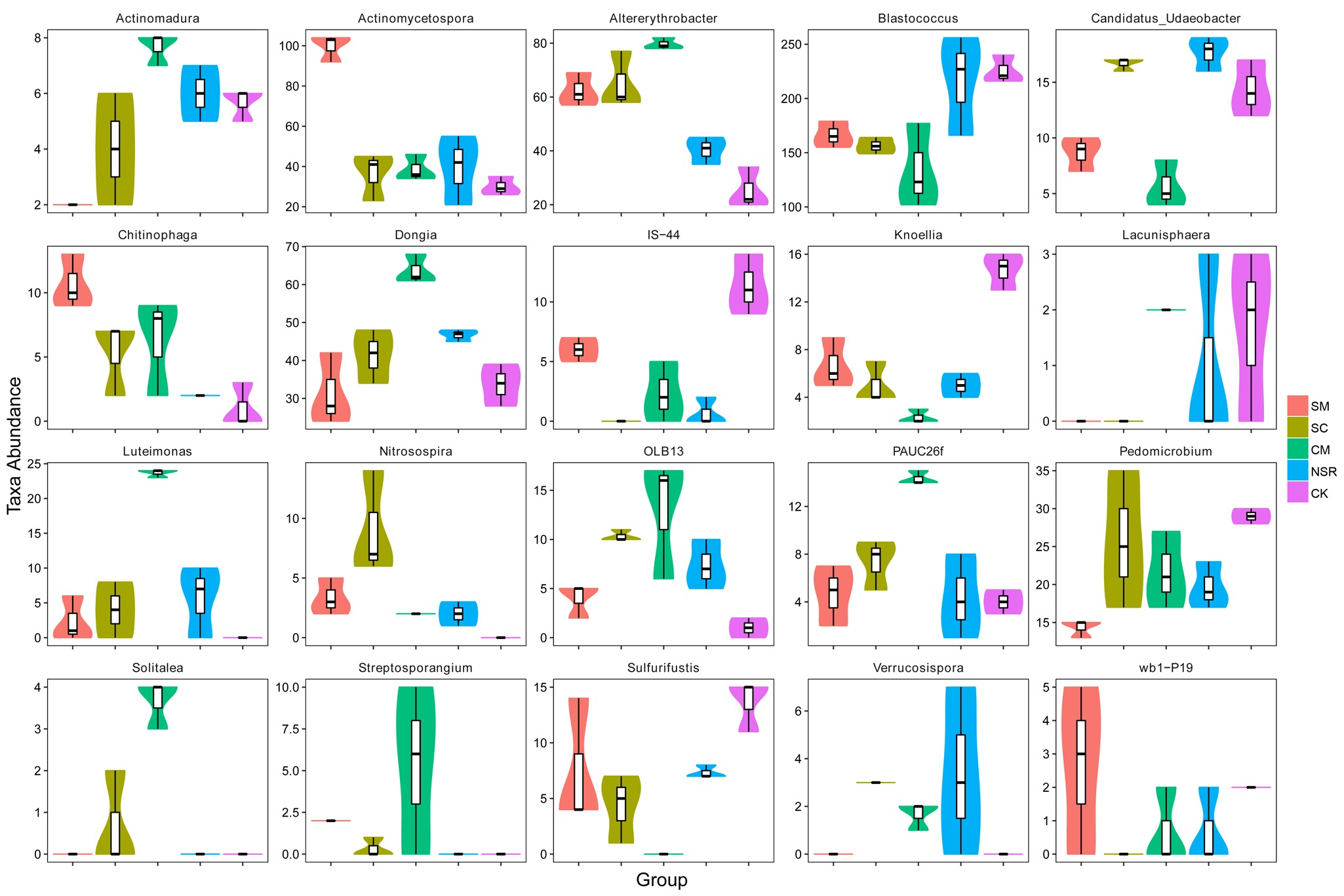

Supplement: S3 Fig — (TIF) [file pone.0249884.s003.tif]

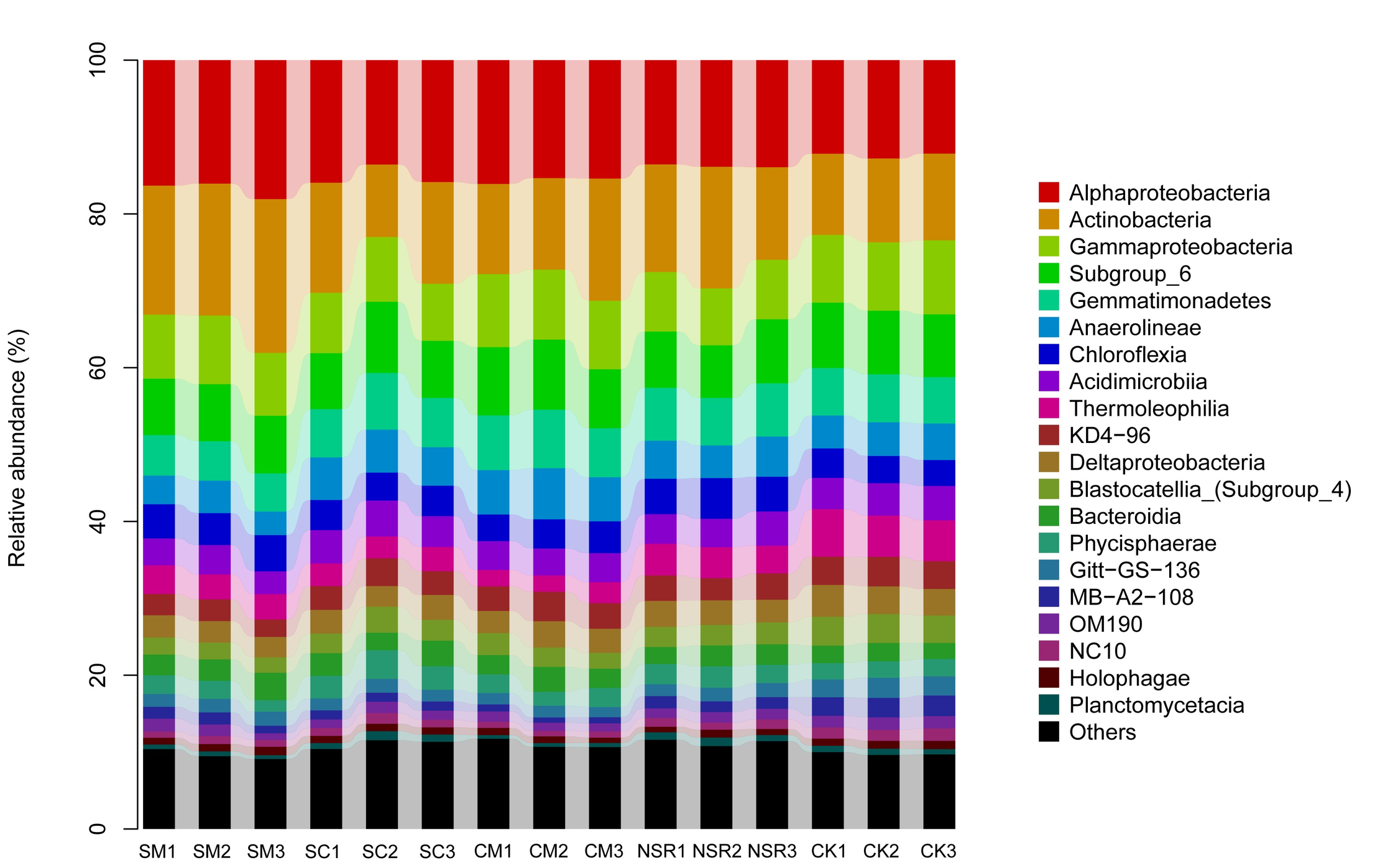

Supplement: S4 Fig — (TIF) [file pone.0249884.s004.tif]

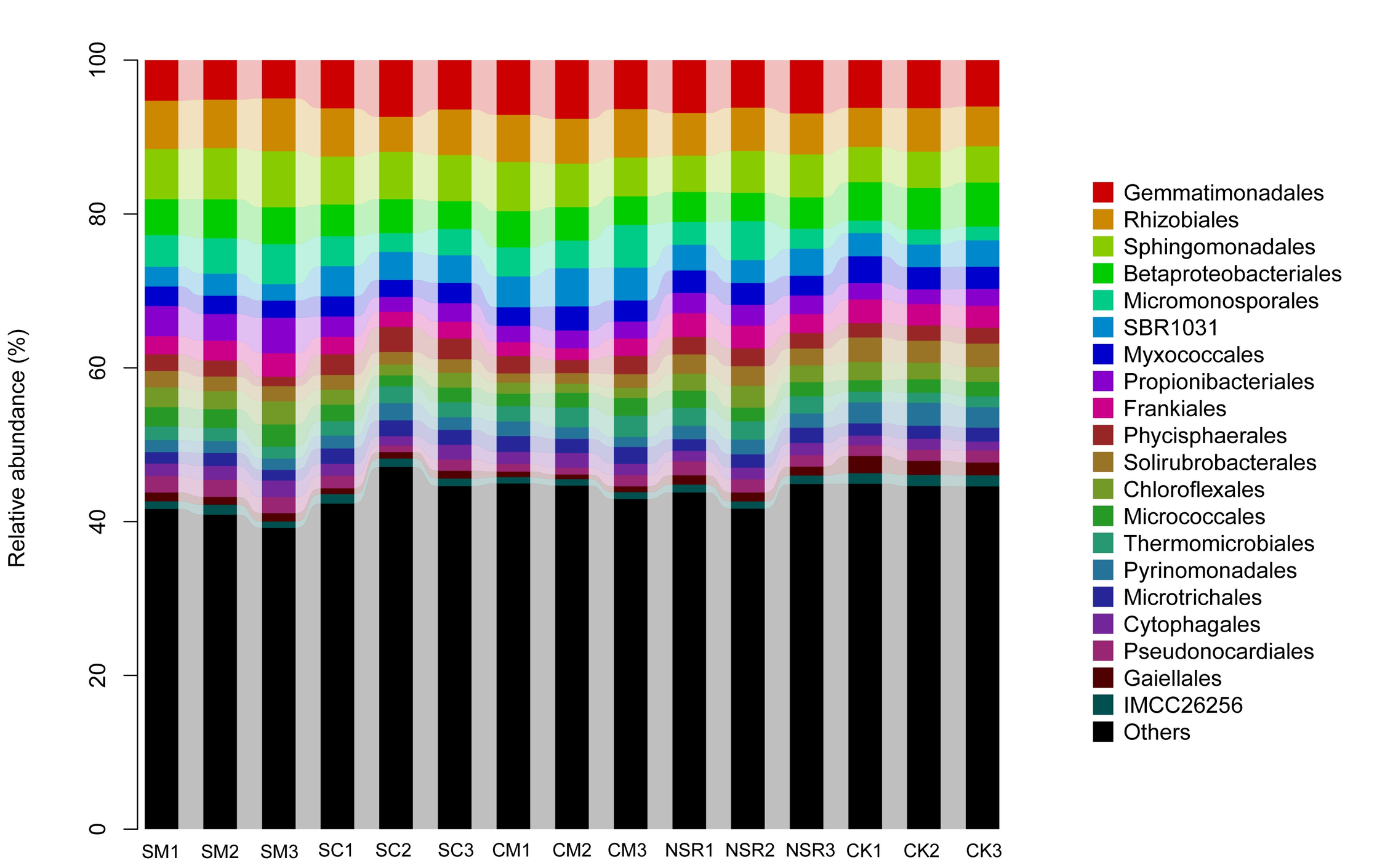

Supplement: S5 Fig — (TIF) [file pone.0249884.s005.tif]

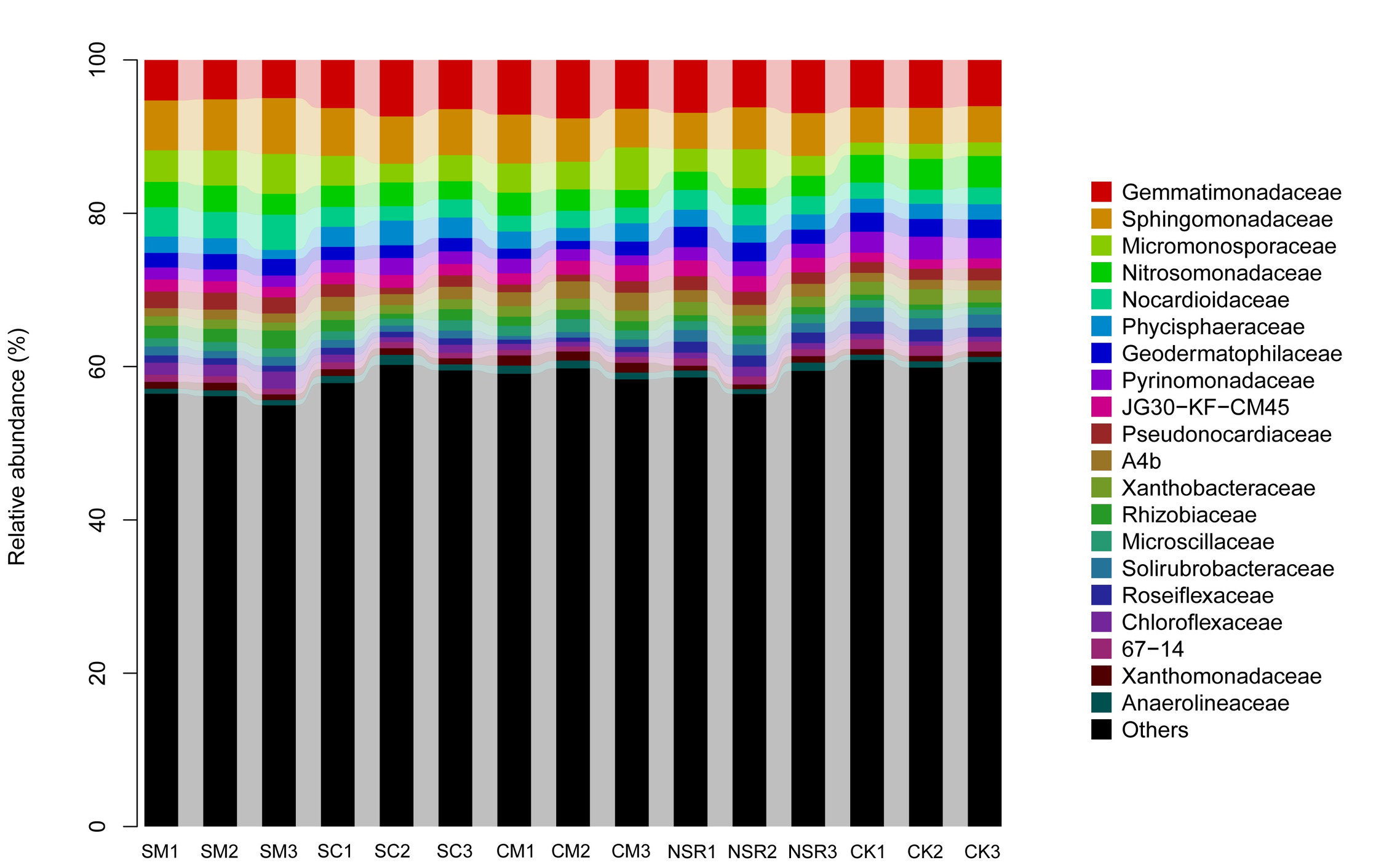

Supplement: S6 Fig — (TIF) [file pone.0249884.s006.tif]

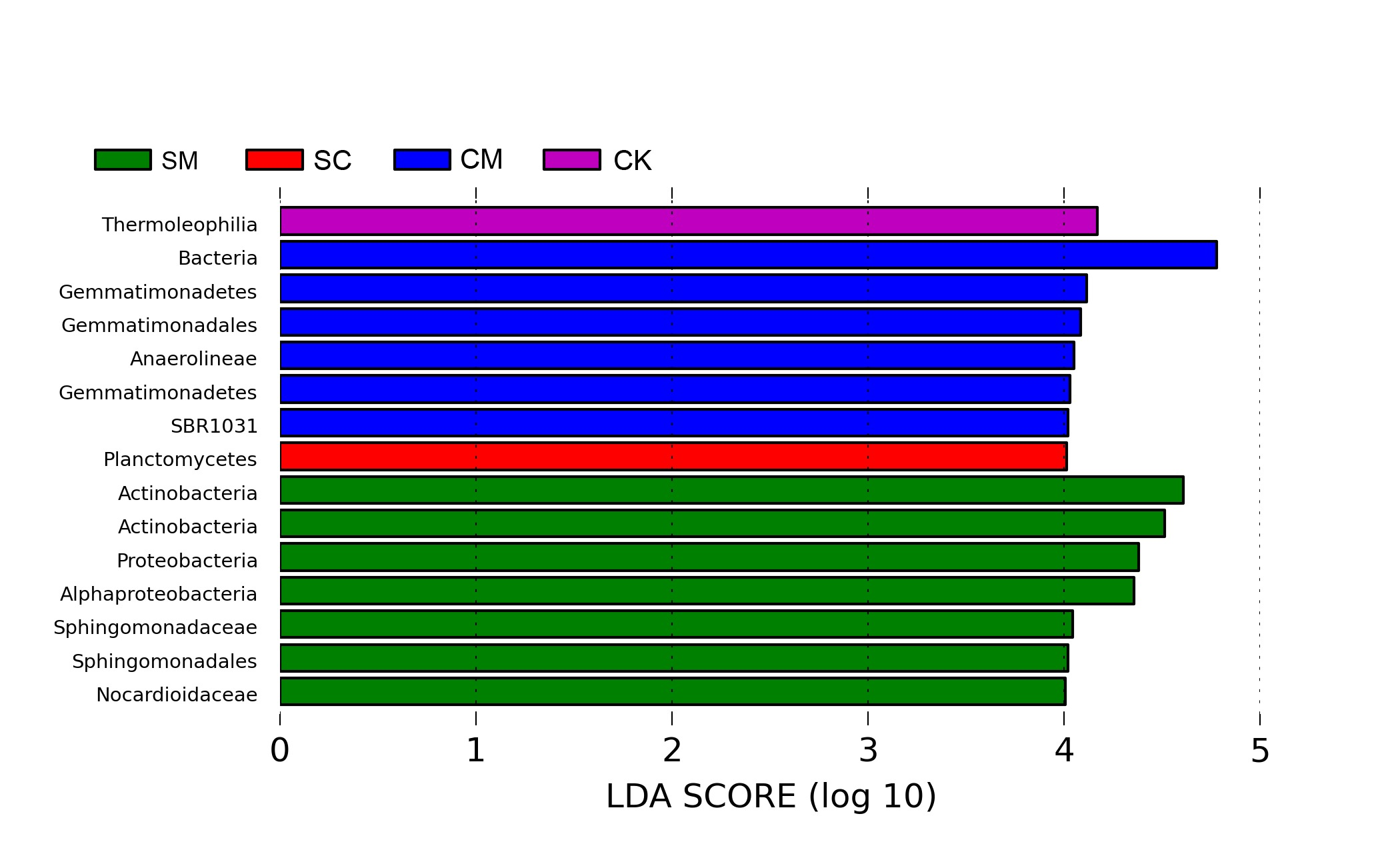

Supplement: S7 Fig — (TIF) [file pone.0249884.s007.tif]
