# Supplementary material for: Long-term effects of maize straw return and manure on the microbial community in cinnamon soil in Northern China using 16S rRNA sequencing
Source: PLoS One. 2021 Apr 22;16(4):e0249884. doi: 10.1371/journal.pone.0249884 (PMC8062091; doi:10.1371/journal.pone.0249884)
Supplement: S1 Table — (DOCX) [file pone.0249884.s008.docx]

S1 Table. The Monte Carlo permutation test

|  | RDA1 | RDA2 | r^2^ | Pr(>r) |  |
| --- | --- | --- | --- | --- | --- |
| pH | -0.90103 | 0.43376 | 0.9062 | 0.001 | *** |
| AN | 0.72884 | -0.68469 | 0.8975 | 0.001 | *** |
| AP | 0.59631 | -0.80276 | 0.8939 | 0.001 | *** |
| AK | 0.38851 | -0.92144 | 0.9390 | 0.001 | *** |
| OM | 0.28167 | -0.95951 | 0.9029 | 0.001 | *** |
| TN | 0.52833 | -0.84904 | 0.9436 | 0.001 | *** |
| TP | 0.42851 | -0.90354 | 0.9310 | 0.001 | *** |
| TK | 0.6746 | -0.73818 | 0.7460 | 0.001 | *** |

# OM: Organic matter, TN: Total N, TP: Total P, TK: Total K, AN: Available N, AP: Available P, AK: Available K. SM: straw mulched; SC, straw crushed; CM, cattle manure; NSR, fertilizer with no straw return; and CK, no fertilizer and no straw return.

Signif. codes: 0 ‘***’ 0.001 ‘**’ 0.01 ‘*’ 0.05 ‘.’ 0.1 ‘ ’ 1

Permutation: free

Number of permutations: 999
